# Reversible Redox Activity by Ion-pH Dually Modulated Duplex Formation of i-Motif DNA with Complementary G-DNA

**DOI:** 10.3390/nano8040226

**Published:** 2018-04-08

**Authors:** Soyoung Chang, Tugba Kilic, Chang Kee Lee, Huseyin Avci, Hojae Bae, Shirin Mesbah Oskui, Sung Mi Jung, Su Ryon Shin, Seon Jeong Kim

**Affiliations:** 1 Center for Bio-Artificial Muscle and Department of Biomedical Engineering, Hanyang University, Seoul 04763, Korea; ssoii88@kaist.ac.kr; 2 Division of Engineering in Medicine, Department of Medicine, Brigham and Women’s Hospital, Harvard Medical School, Cambridge, MA 02139, USA; tugba.kilic@epfl.ch (T.K.); huseyin.avci44@gmail.com (H.A.); shiriinn@gmail.com (S.M.O.); 3 Harvard-MIT Division of Health Sciences and Technology, Massachusetts Institute of Technology, Cambridge, MA 02139, USA; 4 Department of Biomedical Engineering, Izmir Katip Celebi University, Izmir 35620, Turkey; 5 Korea Packaging Center, Korea Institute of Industrial Technology, Bucheon 14449, Korea; withs@kitech.re.kr; 6 Department of Metallurgical and Materials Engineering, Eskisehir Osmangazi University, Eskisehir 26040, Turkey; 7 KU Convergence Science and Technology Institute, Department of Stem Cell and Regenerative Biotechnology, Konkuk University, Seoul 05029, Korea; hojaebae@gmail.com; 8 Future Environmental Research Center, Korea Institute of Toxicology, Jinju 52834, Korea; sungmijung@gmail.com

**Keywords:** carbon nanotube, DNA, biosensor

## Abstract

The unique biological features of supramolecular DNA have led to an increasing interest in biomedical applications such as biosensors. We have developed an i-motif and G-rich DNA conjugated single-walled carbon nanotube hybrid materials, which shows reversible conformational switching upon external stimuli such as pH (5 and 8) and presence of ions (Li^+^ and K^+^). We observed reversible electrochemical redox activity upon external stimuli in a quick and robust manner. Given the ease and the robustness of this method, we believe that pH- and ion-driven reversible DNA structure transformations will be utilized for future applications for developing novel biosensors.

## 1. Introduction

In recent years, DNA has received significant attention due to the advances in structural DNA nanotechnology that have extended its use for development of dynamic and stimuli-responsive nanomaterials in the form of DNA origami, nanomachines, or nanomotors [1,2,3]. Two- or three-dimensional DNA supramolecular structures [4] can demonstrate reversible conformational switching behavior upon external stimuli such as pH [5], interactions with small molecules [6] and other DNA strands [7], light [8], electricity [9], and temperature [10]. For example, the i-motif DNA (i-DNA) is a four-stranded secondary DNA structure, which shows reversible conformational switching at different pH conditions with rapid response time and high cycling stability [11]. The unique biological features of these supramolecular DNA structures have led to an increased interest in the area and resulted in the development of the first pH-driven DNA molecular motor or sensor [12]. On the other hand, G-quadruplex secondary structures, which are complementary to i-DNA, comprise at least two stacks of four guanines by forming specific guanine-rich DNA or RNA molecules that have biological significance [13]. Due to the cation core located at the center, this structure can interact with negatively charged phosphate backbones and remain neutral in charge [14]. These conformational changes may result in unusual structures demonstrating certain advantages, particularly high sensitivity to chemical stimuli such as pH and ions [15]. In addition, hemin/G-quadruplex, a hemin-complexed form of G-quadruplex, was found to act as a horseradish peroxidase (HRP)-mimicking DNAzyme and is hence considered to be a robust and promising platform for biosensor development to detect proteins as well as DNA (Int. J. Electrochem. Sci., 10 (2015) 3897–3913) [16]. 

In this work, we used carbon nanotubes (CNT) for their electrochemical sensing abilities (owing to their unique features, such as conductivity) and monitored unique conformational changes of i-DNA and G-quadruplex as a response to changes in pH and two types of ions. CNT is a uniquely suitable candidate for electrode material in this application because of its high electrical and mechanical properties and its tendency to covalently bond via –COOH or –NH_2_ functional groups [17,18]. In our previous work, a reversible conformational switching of i-DNA alone using changes in pH was demonstrated to induce significant redox activity [19]. In this paper, we demonstrate a reversible redox activity by dually modulated (changes in pH and two types of ions) duplex formation of i-DNA with complementary G-quadruplex. To achieve this, i-DNA and G-quadruplex were first covalently bonded to the surface of the CNT to achieve stable immobilization. Then, reversible conformational switching of the i-DNA and G-quadruplex hybrid by changing pH and lithium (Li^+^) and potassium (K^+^) ion concentrations was evaluated.

## 2. Materials and Methods 

The single walled carbon nanotubes (SWNT) were purchased from CNI Inc. (Madison Heights, MI, USA). DNA with the sequence H_2_NC_6_H_12_–5′–CCCTAACCCTAACCCTAACCCTAA–3′–C_6_H_12_(CH_2_OH)NH_2_ (i-motif DNA) and with the sequence H_2_NC_6_H_12_–5′–GGGTTAGGGTTAGGGTTAGGGTTA–3′–C_6_H_12_(CH_2_OH)NH_2_ (G-quadruplex) were purchased from Integrated DNA Technologies (San Jose, CA, USA). The SWNT was mixed with a solution of sulfuric (H_2_SO_4_) and nitric (HNO_3_) acids in a 3:1 ratio, and sonicated for 1 h in a procedure explained in previously published work to produce functionalized SWNT (f-SWNT) [19]. The f-SWNT was filtered and washed with deionized water. The f-SWNT was sonicated in the presence of EDC/NHS for 30 min. Then, amino-modified oligonucleotides were added and the pH was raised to 6.5. The reaction mixture was stirred for 24 h at room temperature. The reaction mixture was centrifuged for 20 min at 4400 rpm. This solution was purified by dialysis for 6 days at room temperature to remove any unreacted i-motif DNA. The molecular weight cutoff (MWCO) of the membrane was 12,000–14,000 Da. 

Raman spectroscopy was performed using a Jobin Yvon LabRam Model HR800 Raman microscope (Jobin Yvon, Palaiseau, France) equipped with an Argon-ion excitation laser (λ = 514.532 nm). UV-Visible (UV-Vis) spectroscopy was conducted using a Varian UV/VIS/NIR spectrometer (Santa Clara, CA, USA). The structure of i-motif DNA/f-SWNT and G-quadruplex/f-SWNT mixture was examined using SEM (Hitachi Model S4700, Tokyo, Japan) and a High Resolution TEM (Philips CN30, Hillsboro, OR, USA). All Circular Dichroism spectra were recorded on a Jasco-810 spectropolarimeter (Jasco Spectroscopic Co. Ltd., Tokyo, Japan) equipped with a programmable temperature control unit. A three-electrode electrochemical cell coupled to a CHI 600B potentiostat (Austin, TX, USA) was used for the cyclic voltammetry. An i-motif DNA/f-SWNT and G-quadruplex/f-SWNT hybrid electrode was prepared by drop casting of hybrid solution on a glassy carbon electrode and then incubated for 3 days to avoid fall off on the glassy carbon electrode. The i-motif DNA/f-SWNT and G-quadruplex/f-SWNT hybrid electrode was used as the working electrode with an Ag/AgCl reference electrode and a Pt wire counter electrode.

## 3. Results

**Figure 1:**
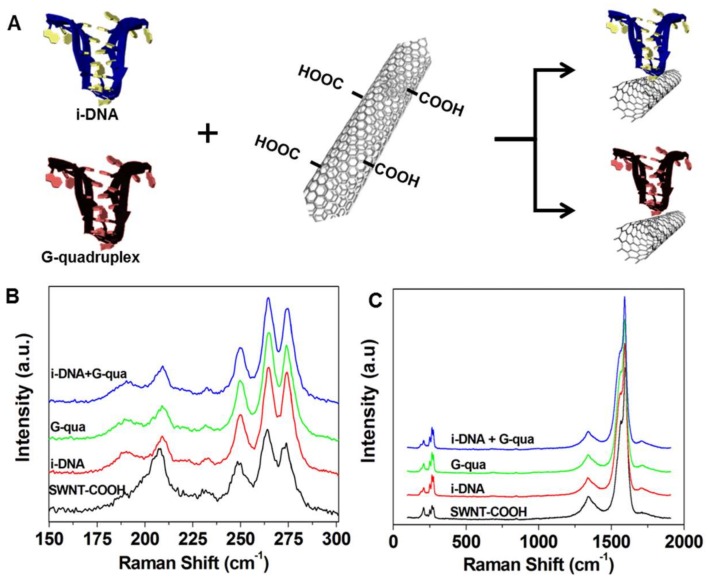
(**A**) Schematic of functionalization of i-motif DNA (i-DNA) and G-quadruplex on f-SWNT; (**B**) Radial breathing mode (RBM) region and (**C**) G and D band Raman spectra of pristine f-SWNT (black), i-DNA/f-SWNT (red), G-quadruplex/f-SWNT (green), and mixture of i-DNA/f-SWNT and G-quadruplex/f-SWNT (blue).

Figure 1A demonstrates the schematic representation of the COOH terminal region on single-walled carbon nanotubes (f-SWNT) attached to i-DNA and G-quadruplex by covalent bonding via *N*-hydroxysuccinimide/*N*-ethylcarbodiimide crosslinking reagent. Raman Spectroscopy was used to characterize the i-DNA/f-SWNT and G-quadruplex/f-SWNT hybrids. As a result, four bands in the radial breathing mode (RBM) frequency region of the Raman spectra (150–300 cm^−1^)—which is known to shift upon modification of i-DNA, G-quadruplex, and i-DNA + G-quadruplex complex [20]—were observed (Figure 1B). These shifts indicate the interactions between DNA structures and f-SWNT. Since RBM frequency is a direct measure of nanotube diameter, the upshift in the peaks is correlated to a decrease in the f-SWNT diameter, indicating a homogenous dispersion of f-SWNT in i-DNA and/or G-quadruplex solution and strong interactions between these molecules [21]. As shown in Figure 1C, three peaks were found in the Raman spectra of i-DNA + G-quadruplex/f-SWNT (blue), G-quadruplex/f-SWNT (green), i-DNA/f-SWNT (red), and pristine f-SWNT (black) dispersions in the 100–2000 cm^−1^ range, which could be attributed to G^+^ band (1590 cm^−1^), G band of the stretching mode (1561 cm^−1^), and D bands that indicate structural defects (1308 cm^−1^) [20]. The G/D ratio of f-SWNT suggests a very low amount of defects and high conductivity in the structure [22]. This ratio is not affected by i-DNA and/or G-quadruplex modification of f-SWNT, which ultimately demonstrates the retained conductive property of f-SWNT.

**Figure 2:**
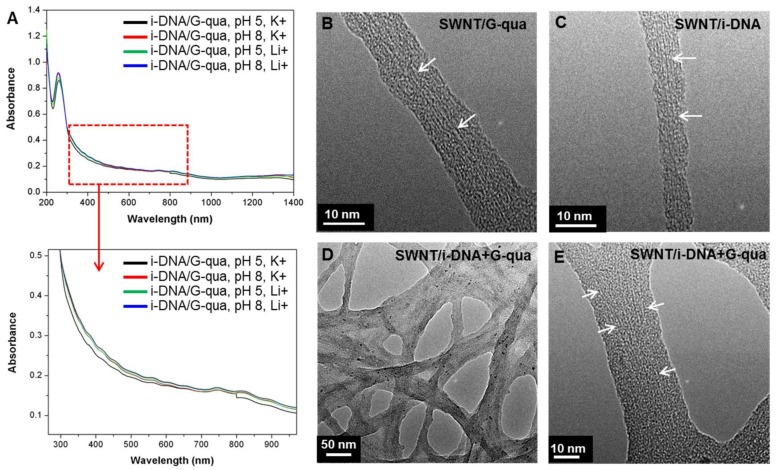
(**A**) UV-Vis absorbance spectra of mixture of i-DNA/f-SWNT and G-quadruplex/f-SWNT at pH 5 and 8 with 100 mM K^+^ and Li^+^. TEM images of (**B**) G-quadruplex/f-SWNT; (**C**) i-DNA/f-SWNT; and (**D**,**E**) mixture of i-DNA/f-SWNT and G-quadruplex/f-SWNT. White arrows indicate the f-SWNT in the hybrids.

To assess the electronic properties of the SWNT in the hybrids, absorbance spectra of i-DNA+G-quadruplex/f-SWNT hybrids at pH 5 and 8 with Li^+^ and K^+^ concentrations of 100 mM were measured (Figure 2A). The absorption peaks presented in Figure 2A show broadened E_11S_, E_22S_, and E_11M_ electronic transitions between pairs of van Hove singularity points in the density of semiconducting and metallic nanotube states, respectively, as a result of interactions between tubes that form into bundle-like structures [20,22]. In order to further analyze the structures, Transmission Electron Microscopy (TEM) images of the SWNTs bound by i-DNA and/or G-quadruplex (Figure 2B–E) were captured. Figure 2B–E show magnified TEM images of f-SWNT bound by G-quadruplex (Figure 2B), i-DNA (Figure 2C), and i-DNA+G-quadruplex (Figure 2D,E). The diameter of SWNT changes throughout the tube axis due to binding of DNA (Figure 2C) and the SWNT are uniformly dispersed (Figure 2D,E). 

**Figure 3:**
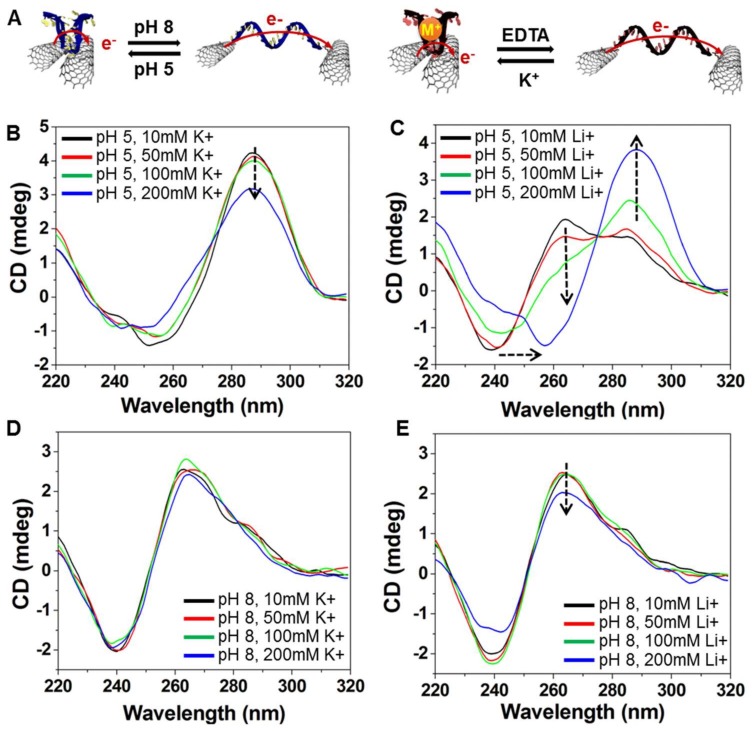
(**A**) Schematic representation of conformational change in response to pH and ion (M^+^) stimuli on i-DNA/f-SWNT (left side) and G-quadruplex/f-SWNT (right side); (**B**–**E**) Circular Dichroism (CD) spectra of a mixture of i-DNA/f-SWNT and G-quadruplex/f-SWNT at various concentrations (10, 50, 100, and 200 mM) of K^+^ and Li^+^ ions in pH 5 (**B**,**C**) and pH 8 (**D**,**E**).

To assess the conformational change in the i-DNA and G-quadruplex structures, Circular Dichroism (CD) spectra of f-SWNT hybrids were obtained at pH values of 5 and 8 and K^+^ or Li^+^ ion concentrations of 10, 50, 100, and 200 mM. Depending on the governing mechanism (i.e., protonation or deprotonation), the change in structure of i-DNA and G-quadruplex resulted in different ellipticity. In a previous study, Kim et al. demonstrated that Li^+^ ions caused destabilization of i-DNA folding structures [23]. The authors used various concentrations of Li^+^ ions (30, 50, 100, 200, or 500 mM) at three different pH values—6.0, 6.2, and 6.4. The folding rate of i-DNA decreases with increasing Li^+^ concentration; however, a low concentration of Li^+^ ions (<200 mM) has a marginal effect on the destabilization of the i-motif under low-pH conditions (pH 6). In addition, Kim et al. demonstrated that Li^+^ ions promote unfolding of the i-motif but do not hinder its folding. In our study, we used a relatively low pH of 5. Although a relatively low concentration of Li^+^ in the environment (<200 mM) induced the destabilization of the i-motif folding formation, this destabilization behavior could be impeded by simply perturbing the surrounding ionic balance using an increased concentration of protons in mediating the hydrogen bonding of cytosine pairs (folding). As illustrated in Figure 3A, acidic conditions favor the formation of four-stranded C-rich i-motifs and C+GC triplets due to deprotonation of cytosines. Likewise, the addition of K^+^ and Li^+^ ions results in a pinched structure for i-DNA but only the K^+^ ion can fold G-quadruplex into a four-stranded structure [24]. In the presence of K^+^ or Li^+^, G-rich sequences form a single-stranded structure. Since the addition of chelator ethylenediaminetetraacetic acid (EDTA) to phosphate buffer solution (PBS) enables the removal of ions, the effect of K^+^ on the conformational change of DNA is reversed. Figure 3B,C show the effect of different concentrations of K^+^ and Li^+^ ion at pH 5 on the conformational change of C-rich and G-rich DNAs. The positive band near 287 nm indicates the folding i-motif and G-quadruplex structures and shows the gradual decrease in ellipticity upon increasing K^+^ ion concentration. The band near 260 nm, on the other hand, shows the existence of single-stranded random coil structures of DNA that can be seen as a shoulder of the latter peak for Li^+^ concentrations of less than 100 mM, which is not enough to fold i-DNA. Moreover, at pH 8, i-motif and G-rich DNA structures adopt random coils and the addition of K^+^ or Li^+^ marginally affects the folding of DNA structures into four-stranded conformations (Figure 3D,E). These results were further proved by Mao et al., where the conformational change of the i-motif and G-quadruplex mixture between folded and unfolded states upon ionic (100 mM Li^+^ and K^+^) or pH-based stimuli (pH 7.4 and pH 5.5) is shown [25].

**Figure 4:**
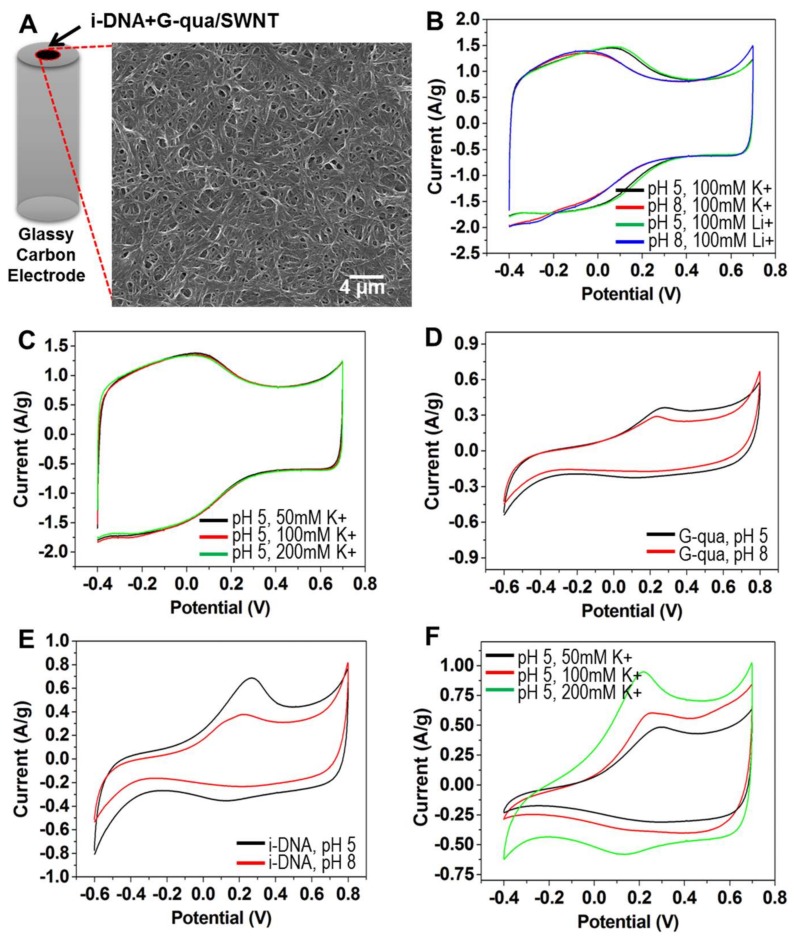
(**A**) Scanning Electron Microscopy (SEM) image of i-DNA/f-SWNT and G-quadruplex/f-SWNT mixture showing fibrous nanostructures, and cyclic voltammetry (CV) curves of (**B**) pristine f-SWNT at pH 5 (black and green) and 8 (red and blue) with 100 mM K^+^ and Li^+^ ions; (**C**) pristine f-SWNT in pH 5 with presence of 50 mM (black), 100 mM (red), and 200 mM (green) K^+^; (**D**) G-quadruplex/f-SWNT in PBS at pH 5 (black) and 8 (red); (**E**) i-DNA/f-SWNT in PBS at pH 5 (black) and 8 (red); and (**F**) mixture of i-DNA/f-SWNT and G-quadruplex/f-SWNT in pH 5 with 50 mM (black), 100 mM (red), and 200 mM (green) K^+^.

To demonstrate the switchable redox activity of i-DNA and G-quadruplex/f-SWNT hybrids, cyclic voltammetry (CV) measurements were performed under various conditions. The morphology of the i-DNA and G-quadruplex/f-SWNT hybrids showed random nanofibrous mesh structures which provide electrically conductive networks (Figure 4A). In Figure 4B,C, pristine f-SWNT showed a rectangular-shaped CV curve that indicated the high electrical conductivity under various conditions. However, the CV curves of pristine f-SWNT were not significantly affected by changing the type of ion at the same pH condition even though the broad and weak redox peaks were shifted by changing pH (Figure 4B). In addition, there are no significant differences in the CV curves of pristine f-SWNT from changing to 150 mM concentrations of K^+^ ions at pH 5 (Figure 4C). As shown in Figure 4D, a pair of redox peaks was observed on G-quadruplex/f-SWNT at both pH 5 and 8 without significant change. Because G-quadruplex has an unfolded structure in both pH 5 and 8 conditions, the redox activity was associated with the bonding of the phosphate groups or nucleobases. However, the i-DNA/f-SWNT hybrid showed significantly different redox activity under changing pH conditions due to the folding of i-DNA which facilitates charge transfer owing to p-stacked hydrogen bonding [26]. As a result, we conclude that folding of i-DNA increases the charge transfer resistance more than does unfolding of G-quadruplex (Figure 4D,E). The effect of different concentrations of K^+^ ions at pH 5 on a mixture of i-DNA/f-SWNT and G-quadruplex/f-SWNT was also investigated (Figure 4F). The CVs at 200 mM K^+^ concentration (green) exhibit a well-defined redox reaction with the highest peak current. In comparison with the electrochemical performance of 50 (black) and 100 (red) mM K^+^ concentrations, smaller changes were observed due to incomplete folding of both G-quadruplex and i-DNA compared with at 200 mM K^+^.

**Figure 5:**
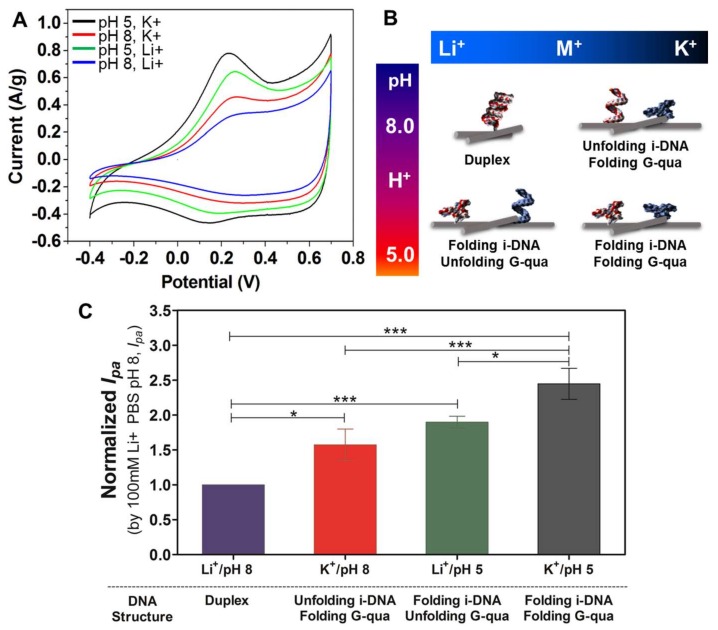
(**A**) Cyclic voltammograms for acidic (pH 5) and basic (pH 8) conditions in the presence of 100 mM either K^+^ or Li^+^ and (**B**) schematic representation of structural transformation of i-DNA/G-quadruplex between folded and unfolded states upon ionic or pH-based stimuli; (**C**) Bar graphs with error bars (*n* = 5) representing the normalized anodic peak current (Ipa) of f-SWNT hybrids either with i-DNA or G-quadruplex at pH 5 (Green/Li^+^ and Grey/K^+^) and pH 8 (purple/Li^+^ and red/K^+^). Annotations *, **, and *** correspond to differences with *p* values less than 0.05, 0.005, and 0.0005, respectively.

Figure 5A shows the CV curves scanned in different solutions with pH 5 (black and green) and 8 (red and blue) of 100 mM K^+^ and 100 mM Li^+^ containing i-DNA/G-quadruplex/f-SWNT hybrids. The highest electrochemical performance is obtained with the hybridization of i-DNA/G-quadruplex/f-SWNT in pH 5 with the presence of 100 mM K^+^ (black). This is due to the folding of both i-DNA and G-quadruplex which results in improved charge transfer. Comparatively, the unfolding of G-quadruplex in pH 5 with 100 mM Li^+^ (green) results in reduction of the charge transfer ability and a lower electrochemical performance. Figure 5B demonstrates a schematic representation of the structural transformations of both i-motif and G-quadruplex DNA structures upon changes of pH or in the presence of various types of ions. As highlighted earlier, pH is an essential factor in determining the structure of i-DNA when Li^+^ ions are present. G-quadruplex structures, on the other hand, are only affected when K^+^ ions are present and do not experience a transformation when Li^+^ is present. Figure 5C demonstrates the normalized peak current of i-DNA/G-quadruplex/f-SWNT hybrids at pH 8 (purple and red) and 5 (green and grey) with solutions containing 100 mM of Li^+^ or K^+^. As demonstrated here, the peak current observed under basic conditions of pH 8 is significantly lower than those observed under more acidic conditions at pH 5. This phenomenon demonstrates that the complementation between duplex formations has prohibited complete folding hybridization of the structures. In addition, the distances of the electrically conductive networks of f-SWNT in hybrids might affect the electrical conductivity and redox properties by quadruplex or duplex structural transformation. In our previous studies, the sizes of the i-motif DNA, G-quadruplex, and i-motif DNA/G-quadruplex mixture (duplex formation) before and after functionalization with carbon nanoparticles (fullerene) in pH 5 and 8 conditions were analyzed using the synchrotron small-angle X-ray scattering technique [27,28,29]. Folded i-motif DNA and G-quadruplex were measured to be ~6 nm and unfolded i-motif DNA and G-quadruplex, and their duplex formation, were measured to be ~8 nm. Therefore, the decreased redox activity and normalized peak current might be induced by the duplex formation of DNAs, resulting in increased distances in the electrically conductive networks of f-SWNT in hybrids under pH 8 with solutions containing 100 mM of Li^+^ conditions.

## 4. Discussion

In summary, systematic experiments were performed to investigate reversible redox activity that responds to changes in pH in the presence of various types of ions using SWNT to illustrate the mechanism of DNA nanomachines. The hybrid structures were obtained by attaching the COOH terminal on SWNT to i-DNA and G-quadruplex. By controlling the two types of ions and pH levels, we can conveniently tune the i-DNA- and G-quadruplex-based switches in a quick and robust manner which can be used as a label-free DNA sensor platform. Using the proposed platform, not only potassium and lithium ions but also pH changes can be detected repeatedly due to highly reversible conformational changes in the i-motif and G-quadruplex structures. Therefore, we believe that this platform could be a significant step towards pH- and ion-driven reversible DNA structure transformations for future applications in the biomedical field. These applications can include uses such as distinguishing a single mismatched sequence from the complementary one, among others.
